# Saturation-Induced Phase Error Compensation Method Using Complementary Phase

**DOI:** 10.3390/mi14061258

**Published:** 2023-06-16

**Authors:** Yingying Wan, Yiping Cao, Min Xu, Tao Tang

**Affiliations:** 1 School of Physical Science and Technology, Southwest Jiaotong University, Chengdu 610031, China; 2 Department of Opto-Electronics, Sichuan University, Chengdu 610064, China; ypcao@scu.edu.cn

**Keywords:** three-dimensional measurement, fringe projection profilometry, phase-shifting, intensity saturation, phase error reduction

## Abstract

Intensity saturation can induce phase error and, thus, measurement error in fringe projection profilometry. To reduce saturation-induced phase errors, a compensation method is developed. The mathematical model of saturation-induced phase errors is analyzed for *N*-step phase-shifting profilometry, and the phase error is approximately *N*-folder of the frequency of the projected fringe. Additional *N*-step phase-shifting fringe patterns with initial phase-shift *π*/*N* are projected for generating a complementary phase map. The final phase map is obtained by averaging the original phase map extracted from the original fringe patterns and the complementary phase map, and then the phase error can be canceled out. Both simulations and experiments demonstrated that the proposed method can substantially reduce the saturation-induced phase error and realize accurate measurements for a highly dynamic range of scenarios.

## 1. Introduction

Three-dimensional (3D) information has found wide applications in online inspection, visual reality (VR) and the protection of historical relics [1,2,3,4]. Fringe projection profilometry (FPP) [5,6,7] as a structured-light (SL) [8]-based method is one of the most representative active 3D measurement technologies owing to its being non-contact, have a high accuracy and a high resolution [9,10,11]. However, due to the limited dynamic range of the camera sensors, the pixels of patterns captured on surfaces with high reflection are prone to be saturated, leading to severe phase errors in FPP.

Scholars have explored many approaches to improve the dynamic range of the images, which are commonly called high dynamic range (HDR) 3D measurement methods. These methods can be mainly classified into two categories: equipment-based techniques and algorithm-based techniques [12]. The equipment-based techniques avoid the saturation of the captured images by adjusting the parameters of the hardware or using additional devices, which can be further divided into three classes: multi-exposure methods [13,14], adaptive projection methods [15,16] and additional equipment methods [17,18]. The multi-exposure methods generally capture a set of images with different exposures, and then fuse them to an HDR image using multi-exposure fusion algorithms [19]. Since multi-exposure methods require taking a large number of images to obtain an image with a high dynamic range, it is very time consuming. In recent years, although some approaches were proposed to improve the measurement efficiency [20,21], their measurement accuracy was sacrificed to some extent. Instead of adjusting the exposure of the camera, the adaptive projection methods change the brightness of the projected images pixel by pixel to obtain the desired image without saturation. However, these methods require intense calibration in advance, which limits their application in dynamic scenarios. Some scholars reduced the effect of high reflective surfaces by introducing additional equipment in a traditional projector-camera FPP system: linear polarizer [22], digital micromirror device (DMD) [23], transparent screen [24], etc. Among the additional equipment methods, polarization-based methods are popularly researched owing to their additional polarization information. Zhu et al. [25] proposed a coding strategy based on polarization and light intensity utilizing the polarization characteristics of a liquid crystal display projector. This method could measure objects with specular reflections and greatly reduced the interference of noise. To further improve the stability of HDR measurements, the same group proposed a polarization-plus-phase-shift coding strategy, and achieved robust phase unwrapping [26]. However, these additional equipment methods had some shortcomings such as reducing the signal-to-noise ratio (SNR) and sacrificing spatial resolution.

The algorithm-based techniques use certain algorithms to derive the phase information by utilizing the saturated fringe patterns without adding or adjusting the hardware equipment. Hu et al. [27] found that the effect of the image saturation can be suppressed in phase-shifting profilometry (PSP) if the phase shift is large enough to ensure that three fringe intensities are not saturated. However, different phase-shifting (PS) algorithms using entirely different phase calculations would increase the complexity of the algorithm. Chen et al. [28] suggested estimating the phase using a standard *N*-step phase-shifting algorithm directly from the saturated phase-shifting patterns. This method could obtain a high-quality phase if there was no phase-shift error, but it required a large number of phase-shifting fringe patterns and was still time-consuming. To reduce the required images, Qi et al. [29] introduced a phase-error theory for a standard *N*-step phase-shifting algorithm, and suggested that a seven-step phase-shifting algorithm could reduce the phase error induced by the intensity saturation. Jiang et al. [30] and Wang et al. [31] achieved high dynamic range 3D measurements using additional inverted three-step PS fringe patterns. These methods used unsaturated inverted fringe patterns to replace saturated original fringe patterns for phase acquisition; however, phase calculation involves different equations according to different saturation degrees. To achieve more generic measurements, Tan et al. [32] proposed a saturation-induced error correction method using joint Fourier analysis and Hilbert transform, which could effectively reduce the phase error based on its periodic characteristic. However, errors would occur at the fringe edges due to the Hilbert transform.

This paper presents a flexible saturation-induced phase error reduction method using a complementary phase. The mathematical model of the saturation-induced phase error for a phase-shifting algorithm is analyzed considering the fifth-order harmonic, and an additional set of phase-shifting fringe patterns with initial phase-shift *π/N* are projected for compensating the phase error. The original phase map and the complementary phase map are calculated from the saturated original fringe patterns and shifted fringe patterns, respectively. The final phase map is generated by averaging two phase maps, and then the saturation-induced phase error can be effectively reduced. Unlike the transform-based methods, the complementary phase map is calculated pixel by pixel for the phase error compensation. As a result, the measurement accuracy of the full-field phase can be improved compared with that of the transform-based methods, which might cause errors at the object edges. Simulation and experimental results show that the proposed method can obtain a high-quality phase map and realize accurate measurements for HDR scenarios.

## 2. Methods

### 2.1. N-Step Phase-Shifting Algorithm

Phase-shifting methods are widely used in structured-light 3D measurements due to their high accuracy and easy implementation. The intensity of the *n*th captured phase-shifting fringe pattern in a standard *N*-step phase-shifting algorithm can be expressed as:

(1) $$I_{n}(x,y)=A(x,y)+B(x,y)\cos[\varphi (x,y)+\delta_{n}],n=1,2,\ldots ,N$$

where (x,y) are the image coordinates, $\delta_{n}=2\pi n/N$ is the phase shift, A(x,y) is the background intensity, B(x,y) is the intensity modulation and φ(x,y) is the phase containing depth information and needs to be solved for. The phase can be calculated using a phase-shifting algorithm when N≥3 [6]:

(2) $$\varphi (x,y)=\arctan\frac{\sum_{n=1}^{N}I_{n}(x,y)\sin\delta_{n}}{\sum_{n=1}^{N}I_{n}(x,y)\cos\delta_{n}}$$

where the arctangent function results in a wrapped phase with 2*π* discontinuities. The continuous phase map Φ(x,y) can be obtained using the wrapped phase φ(x,y) and fringe order k(x,y), which can be determined using a phase unwrapping algorithm [5]:

(3) Φ(x,y)=φ(x,y)+2πk(x,y)

The unwrapped phase can be used for 3D reconstruction once the system is calibrated [33]. As a result, the accuracy of the 3D measurement is mainly determined by the quality of the phase map.

### 2.2. Saturation-Induced Phase Error Analysis

The *N*-step phase-shifting algorithm can derive an accurate phase map if the intensity of the fringe patterns is a sinusoidal distribution. However, the image saturation will cause non-sinusoidal fringes and induce severe phase errors. The non-sinusoidal PS fringe patterns as a result of image saturation can be considered as the aliased signal with different frequencies, and can be modeled as:

(4) $$I_{n}^{s}(x,y)=A(x,y)+\sum_{j=1}^{p}B_{j}(x,y)\cos\left\{j[\varphi (x,y)+\delta_{n}]\right\}$$

where $B_{j}(x,y)$ is the modulation of the *j*th harmonic, and p is the number of the harmonics. When p=1, $I_{n}^{s}(x,y)=I_{n}(x,y)$, which are the ideal sinusoidal fringe patterns. When p>1, the fringe patterns become non-sinusoidal. The measured phase using an *N*-step PS algorithm can be calculated by submitting Equation (4) into Equation (2):

(5) $$\varphi^{s}=\arctan\frac{\sum_{n=1}^{N}I_{n}^{s}\sin\delta_{n}}{\sum_{n=1}^{N}I_{n}^{s}\cos\delta_{n}}$$

Note that in Equation (5) and the remainder of this paper, (*x*, *y*) may be omitted for brevity. The saturation-induced phase error can be calculated by taking the difference between the measured phase $\varphi^{s}$ and the real phase φ:

(6) $$\begin{array}{ll} \Delta \varphi & =\varphi^{s}-\varphi \\ & =\arctan\frac{\sum_{n=1}^{N}\sum_{j=2}^{p}\left(B_{j+1}-B_{j-1}\right)\sin[j(\varphi +\delta_{n})]}{B_{1}N+\sum_{n=1}^{N}\sum_{j=2}^{p}\left(B_{j+1}+B_{j-1}\right)\cos[j(\varphi +\delta_{n})]} \end{array}$$

For the *N*-step PS algorithm, phase errors are sensitive to the order *p* = *jN*, while errors related to harmonics with other orders are suppressed [32]. The saturation-induced phase error can be further described as:

(7) $$\Delta \varphi =\arctan\frac{\sum_{j=1}^{p}\left(C_{jN+1}-C_{jN-1}\right)\sin(jN\varphi )}{1+\sum_{j=1}^{p}\left(C_{jN+1}-C_{jN-1}\right)\cos(jN\varphi )}$$

where constant $C_{k}=B_{k}/B_{1}$. The saturation-introduced phase error highly correlates to the real phase φ. Since 3-step and 4-step phase-shifting algorithms are more extensively used in SL measurements, the phase errors for these two methods were analyzed in detail.

For the 3-step phase-shifting algorithm (i.e., *N* = 3), considering the fifth-order harmonic, the saturation-induced phase error is given as:

(8) $$\Delta \varphi =\arctan\frac{\left(C_{4}-C_{2})\sin(3\varphi )-C_{5}\sin(6\varphi \right)}{1+(C_{4}+C_{2})\cos(3\varphi )+C_{5}\cos(6\varphi )}$$

Considering $C_{k}$ is much smaller than 1, Equation (8) can be approximately expressed as:

(9) $$\Delta \varphi \approx (C_{4}-C_{2})\sin(3\varphi )-C_{5}\sin(6\varphi )$$

In general, the modulation of the harmonics decreases with the harmonics order increasing, and $C_{4}-C_{2}$ is much larger than $C_{5}$. The saturation-induced phase error can be further approximated as:

(10) $$\Delta \varphi \approx (C_{4}-C_{2})\sin(3\varphi )$$

For the 4-step phase-shifting algorithm (i.e., *N* = 4), considering the fifth-order harmonic, the saturation-induced phase error is derived as:

(11) $$\Delta \varphi =\arctan\frac{\left(C_{5}-C_{3})\sin(4\varphi \right)}{1+(C_{5}+C_{3})\cos(4\varphi )}$$

Similarly, the phase error can be approximately described as:

(12) $$\Delta \varphi \approx (C_{5}-C_{3})\sin(4\varphi )$$

The above analysis reveals that for the 3-step and 4-step phase-shifting algorithms, the saturation-induced phase error is approximately *N*-folder of the frequency of the projected fringe patterns. Generally, the saturation-induced phase error for an *N*-step PS algorithm can be approximated as:

(13) $$\Delta \varphi \approx K_{N}\sin(N\varphi )$$

where $K_{N}$ is a constant. It should be noted that the saturation-induced phase error model derived as Equation (13) is consistent with that in Ref. [32]; however, our method does not involve Fourier filtering as Ref. [32] did, which might filter out some detailed information.

### 2.3. Phase Error Compensation Using a Complementary Phase

To reduce the saturation-induced phase error, a compensation method using a complementary phase is introduced. Additional *N*-step phase-shifting fringe patterns with initial phase-shift *π*/*N* are projected onto the measured object. The intensities of the captured shifted fringe patterns are expressed as:

(14) $$I_{n}^{s}{}^{\prime }(x,y)=A(x,y)+\sum_{j=1}^{p}B_{j}(x,y)\cos\left\{j[\varphi (x,y)+\delta_{n}+\pi /N]\right\}$$

Submitting Equation (14) into Equations (5)–(7), the saturation-induced phase error using shifted fringe patterns can be described as:

(15) $$\begin{array}{ll} \Delta \varphi^{\prime } & =\arctan\frac{\sum_{j=1}^{p}\left(C_{jN+1}-C_{jN-1}\right)\sin(jN\varphi +j\pi )}{1+\sum_{j=1}^{p}\left(C_{jN+1}-C_{jN-1}\right)\cos(jN\varphi +j\pi )}\\ & \approx K_{N}\sin(N\varphi +\pi )\\ & \approx -K_{N}\sin(N\varphi ) \end{array}$$

The original phase $\varphi^{s}$ calculated using original saturated phase-shifting patterns and the complementary phase $\varphi^{s}{}^{\prime }$ calculated with additional phase-shifting patterns shifted with *π*/*N* can be expressed as:

(16) $$\begin{array}{l} \varphi^{s}=\varphi +\Delta \varphi =\varphi +K_{N}\sin(N\varphi )\\ \varphi^{s}{}^{\prime }=\varphi +\Delta \varphi^{\prime }=\varphi -K_{N}\sin(N\varphi ) \end{array}$$

Since the phase error of the original phase map and that of the complementary phase map have opposite distributional tendencies, they can be canceled out by averaging these two phase maps. Thus, the final phase φ can be obtained by:

(17) $$\varphi =(\varphi^{s}+\varphi^{s}{}^{\prime })/2$$

## 3. Simulations

Some simulations were carried out to verify the performance of the proposed compensation method for saturation-induced phase error. The intensities of the phase-shifting fringes are encoded as:

(18) $$I_{n}=127.5+127.5\cos[2\pi fx+2\pi n/N]$$

where *f* is the fringe frequency. The captured intensities of the fringe patterns are expressed as:

(19) $$I_{n}^{r}=R\left\{127.5+127.5\cos[2\pi fx+2\pi n/N]\right\}$$

where *R* is the reflectance of the surface. The saturated fringe patterns are described as:

(20) $$I_{n}^{s}=\left\{ \begin{array}{l} I_{n}^{r},\;\;\;\;I_{n}^{r}\leq 255\\ 255,\;I_{n}^{r}>255 \end{array}\right.$$

where 255 is the maximum dynamic range (eight-bit) in this simulation, and the saturation degree can be represented by *R*. 

**Figure 1:**
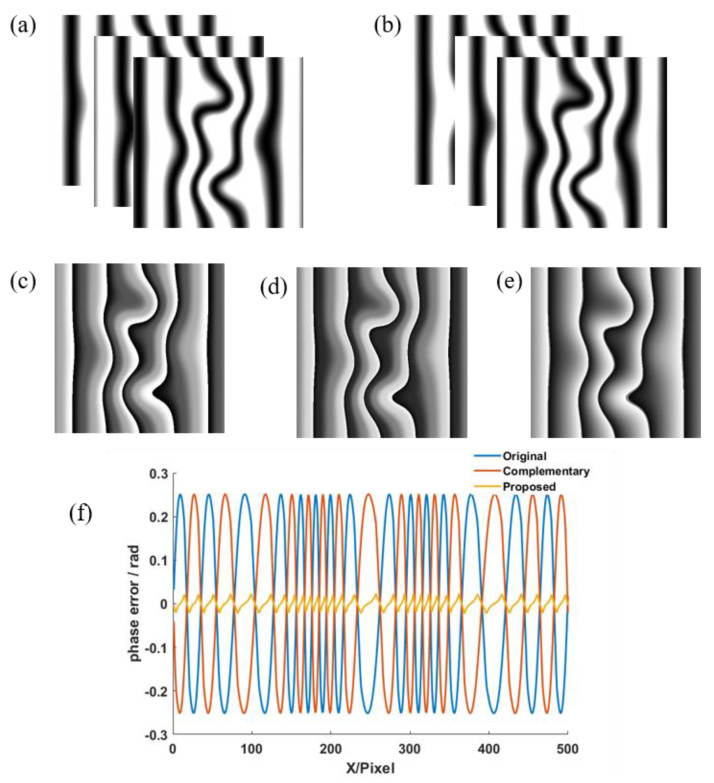
Simulated results: (**a**) original saturated 3-step PS fringe patterns, (**b**) *π*/3 shifted saturated 3-step PS fringe patterns, (**c**) original phase map, (**d**) complementary phase map, (**e**) final phase map, (**f**) phase error cross sections of (**c**–**e**).

The simulated results are shown in Figure 1, where the simulation parameters are *N* = 3 and *R* = 1.5. Figure 1a,b show the original and *π*/*3* shifted saturated three-step phase-shifting fringe patterns. The original phase map calculated using the original fringe patterns and the complementary phase map calculated using the *π*/3 shifted fringe patterns are shown in Figure 1c,d, respectively. The final phase map obtained by averaging the original phase map and the complementary phase map is shown in Figure 1e. To better illustrate the compensation method, phase error cross sections of the original phase map, the complementary phase map and the phase map using the proposed method are shown in Figure 1f. The phase error distributional tendencies of the original phase and the complementary phase are opposite, which verifies the principle in Section 2. It can be seen from Figure 1f that the phase error of the final phase was reduced effectively by using the proposed method.

**Figure 2:**
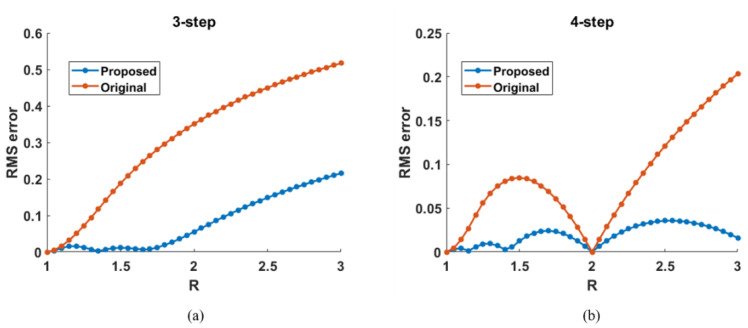
Phase error with different saturation degrees using (**a**) 3-step PS algorithm, (**b**) 4-step PS algorithm.

To quantitatively analyze the phase errors, root-mean-square (RMS) error using three-step and four-step PS algorithms with different saturation degrees (*R* = 1, …, 3) were evaluated, as shown in Figure 2. The phase error using a three-step PS algorithm before compensation increased largely with the increase in the saturation degree, as shown in Figure 2a. In contrast, the phase error after compensation was reduced to a low level when R≤2, and reduced more than half when *R* > 2. The phase error using a four-step PS algorithm reached its minimum when *R* = 2, and it was reduced effectively after the proposed compensation method, as shown in Figure 2b. 

## 4. Experiments and Results

To verify the proposed compensation method for saturation-induced phase error, a FPP system was set up, which consisted of a digital-light-processing (DLP) projector (Wintech PRO4500, Wintech Digital System Technology Corp., San Marcos, CA, USA) with 912 × 1140 resolution, one mono camera (Basler acA2040-120um, Basler AG, Ahrensburg, Germany) with 2048 × 1536 resolution and a 16 mm focal length lens. Since the 3-step PS algorithm is popularly used because of its high efficiency, it was adopted in the following experiments. Additionally, a multi-frequency temporal phase unwrapping algorithm [34], with fringe frequencies of 1, 6 and 20, was used for obtaining the continuous phase map.

### 4.1. Effectiveness Evaluation Measurement

**Figure 3:**
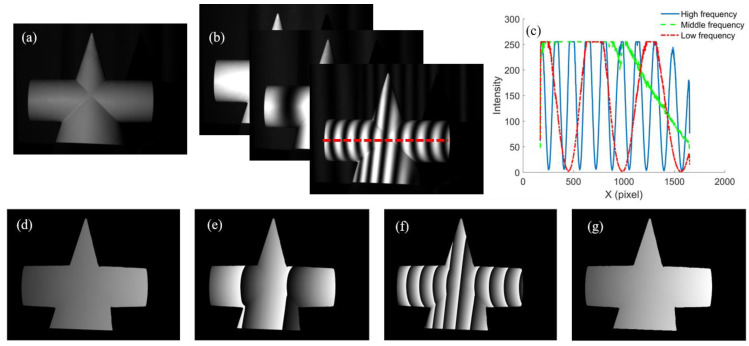
Measurement results of a geometric plaster model: (**a**) image of the geometric plaster; (**b**) captured fringe patterns of three frequencies; (**c**) intensities of the red dashed line in (**b**); wrapped phase map of (**d**) low frequency, (**e**) middle frequency, (**f**) high frequency; (**g**) continuous phase map of high frequency.

In order to verify the effectiveness of the proposed saturation-induced phase error compensation method, experiments were performed on a geometric plaster model as shown in Figure 3a. Figure 3b shows the captured fringe patterns of three frequencies, and the corresponding intensities of the dashed line are shown in Figure 3c, where the fringe intensities were saturated. The wrapped phase maps of the low frequency, middle frequency and high frequency were obtained using the proposed method, and are shown in Figure 3d–f, respectively. The continuous phase map of the high frequency obtained using a temporal phase unwrapping algorithm is shown in Figure 3g. The results verified the proposed method were appropriate for projecting both low and high frequency fringes.

**Figure 4:**
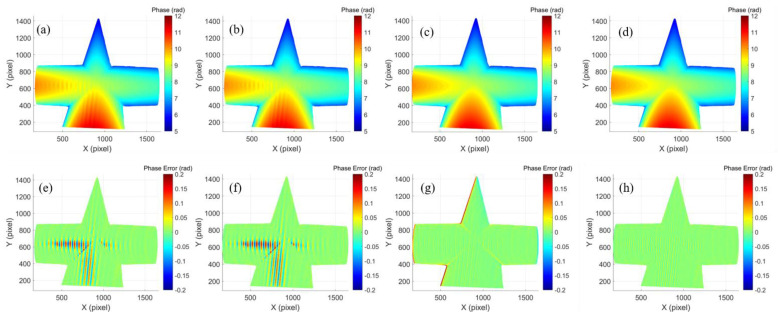
Reconstruction result of a geometric plaster model: phase map using (**a**) original fringe patterns, (**b**) *π*/*N*-shifted fringe patterns, (**c**) method with Fourier filtering, (**d**) proposed method without Fourier filtering, (**e**–**h**) phase error of (**a**–**d**).

Figure 4a shows the phase reconstruction obtained from the original fringe patterns where the saturation-induced error was obvious (i.e., ripples on the surface). Figure 4b shows the phase reconstruction obtained from *π*/*N*-shifted fringe patterns, and the saturation-induced error was also obvious. To verify the phase error model of the proposed method, the captured images with and without Fourier filtering were used for obtaining the phase maps as shown in Figure 4c,d. It demonstrated that both methods with and without Fourier filtering could greatly reduce the measurement error. To quantitatively evaluate the performance of the proposed method, a 16-step phase-shifting algorithm was adopted to obtain the ground truth of the measured object [35]. The phase error maps of phases in Figure 4a–d are shown in Figure 4e–h, respectively. The mean absolute error (MAE) and the RMS error using the original fringe patterns were 0.1879 rad and 0.0254 rad, respectively. The result using *π*/*N*-shifted fringe patterns had a close error distribution: an MAE of 0.2169 rad and an RMS error of 0.0276 rad. The RMS error using Fourier filtering was reduced to 0.0175 rad, while the MAE was increased to 0.4160 rad due to the large errors at the object edges. In contrast, the MAE and the RMS error using the proposed method without Fourier filtering was reduced to 0.0702 rad and 0.0083 rad, respectively. The comparison of the above experimental results is shown in Table 1. The results demonstrated that the proposed method could reduce the phase error effectively in full field and avoid the errors at the object edges.

**Figure 5:**
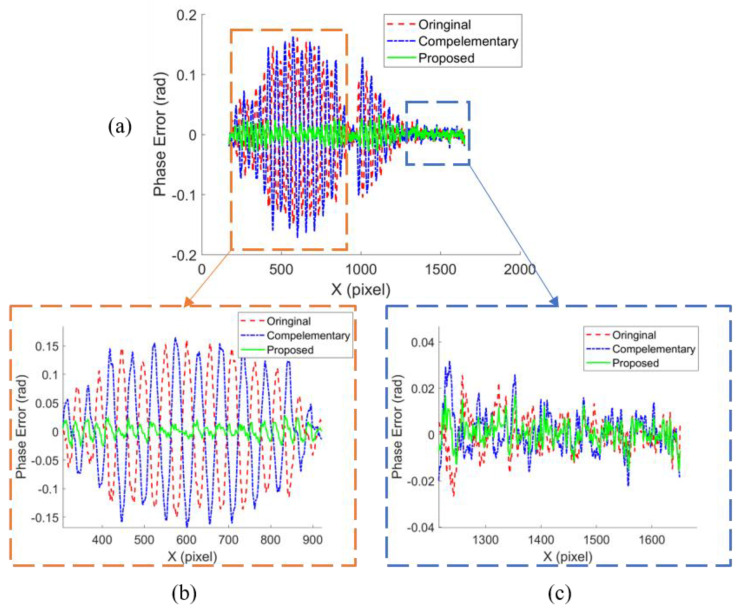
Measurement result: (**a**) phase error of original phase, complementary phase, and proposed phase; (**b**) phase errors in saturated region; (**c**) phase errors in unsaturated region.

The phase error of the original phase, the complementary phase and the proposed phase on the dashed line in Figure 3b are plotted in Figure 5a. The saturated region (in orange dashed box) and the unsaturated region (in blue dashed box) is enlarged in Figure 5b,c, respectively. In the unsaturated region, the phase errors of the above three phase maps had a similar distribution at a relatively low level. In contrast, in the saturated region, the phase error of the original and the complementary phases were opposite and were cancelled out by averaging the phase maps. 

### 4.2. Comparison Measurement of Complex Surface

**Figure 6:**
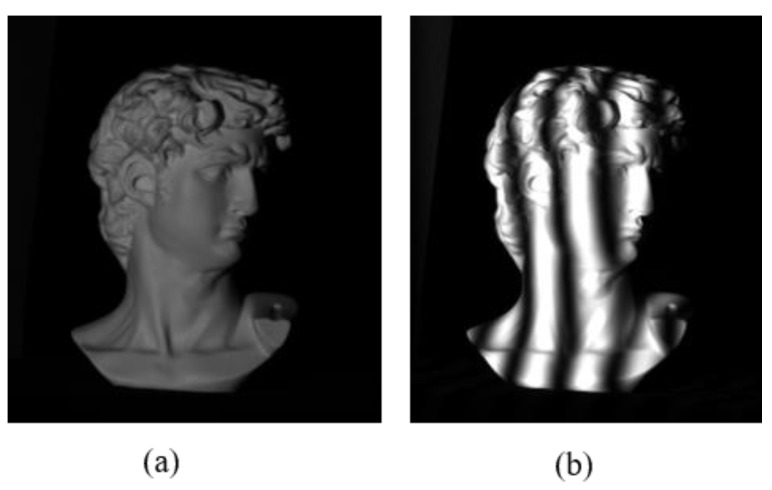
Measurement result of a David model: (**a**) image of the David model, (**b**) one of the captured fringe patterns.

Comparison measurements of a David model with a complex surface were conducted employing Tan’s method [32] and the proposed method. The image of the David model and one of the captured fringe patterns are shown in Figure 6a,b.

**Figure 7:**
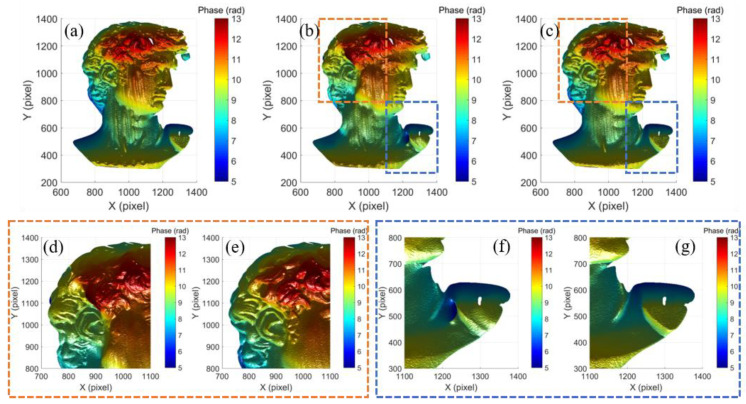
Measurement result of a David model: phase map using (**a**) original fringe patterns, (**b**) Tan’s method, (**c**) proposed method; details of Tan’s method and proposed method: (**d**,**e**) in orange dashed box, (**f**,**g**) in blue dashed box.

The compared experimental results are shown in Figure 7. Figure 7a–c show the phase maps obtained using the original fringe patterns, Tan’s method and the proposed method, respectively. The result using original fringe patterns shows severe saturation-induced error, while both Tan’s method and the proposed method could effectively alleviate the induced error and greatly improve the measurement quality. To give a better illustration of the measurement results of Tan’s method and the proposed method, the details in the dashed regions in Figure 7b,c are enlarged in Figure 7d–g, respectively. There were some residual errors at the hair region and the shoulder region in Tan’s method, as shown in Figure 7d,f, while the proposed method still performed well on the complex surface, as shown in Figure 7e,g.

**Figure 8:**
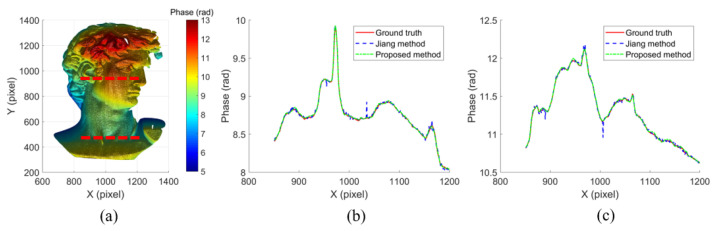
Comparison results of a David model: (**a**) phase map using Jiang’s method, (**b**) cross sections of the lower dashed line in (**a**,**c**) cross sections of the upper dashed line in (**a**).

To further verify the performance of the proposed method, the experiment on the David model was conducted using Jiang’s method [30] for comparison. The phase reconstruction of Jiang’s method is shown in Figure 8a. To be more intuitive, the phase cross sections of the ground truth, Jiang’s method and the proposed method on the lower and upper dashed lines in Figure 8a are shown in Figure 8b,c, respectively. There were some phase jumps in Jiang’s method. In contrast, the results of the proposed method were smooth and closer to the ground truth, which demonstrated that the proposed method was less sensitive to the noise.

### 4.3. Measurements for an HDR Scenario

**Figure 9:**
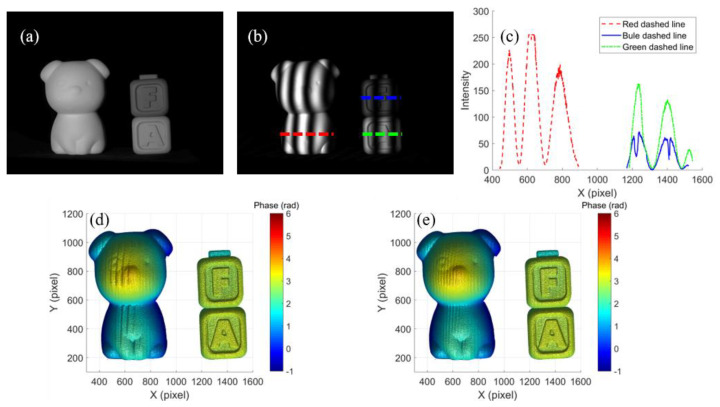
Measurement result of an HDR scenario: (**a**) image of the three measured objects, (**b**) one of the captured fringe patterns, (**c**) the intensity of the dashed line in (**b**), reconstruction: (**d**) using original fringe patterns, (**e**) using the proposed method.

In order to evaluate the performance of the proposed method for an HDR scenario, three objects with different reflectivity were measured. Figure 9a shows the measured objects, one dog model and two letter blocks, and Figure 9b shows one of the captured fringe patterns. The intensity cross sections of the dashed line on the three objects are shown in Figure 9c, and different intensity ranges demonstrated that the objects had different reflectance. The result using the original fringe patterns and the result using the proposed method are shown in Figure 9d,e, respectively. The phase maps were reconstructed successfully, both using the original fringe pattern method and the proposed method, where the fringe patterns were not saturated (on letter blocks). When the intensity saturation occurred, the result using the original fringe patterns suffered from saturation-induced error (on the dog model), while the result using the proposed method had less error. The experimental results showed that the proposed method had a better performance on suppressing saturation-induced errors caused by high reflectivity, and could be used for HDR scenarios.

## 5. Conclusions

A new saturation-induced error compensation method based on complementary phase was presented for phase-shifting profilometry. The saturation-induced phase error model for an *N*-step PS algorithm was mathematically analyzed and optimized considering the fifth-order harmonic, and could be generally approximated as *N*-folder of the frequency of the projected fringes. Consequently, a set of *N*-step phase-shifting fringe patterns with initial phase-shift *π*/*N* were projected to generate a complementary phase pixel by pixel for compensating the phase error. Simulation and Experimental results verified that the proposed method could substantially reduce the phase error induced by the intensity saturation in full field. Compared with the transform-based method, the proposed method with pixel-wise complementary phase could extract higher accurate phases at the object edges and perform better on complex surfaces. Compared with the inverted-fringe method, the compensation method was less sensitive to noise. In addition, the experimental results of the objects with different reflectance demonstrated that highly accurate measurements could be achieved for HDR scenarios using the proposed method.

## Figures and Tables

**Table 1:** Comparison of experimental results for geometric plaster model (rad).

	Method Using Original Fringe Patterns	Method Using *π/N* Fringe Patterns	Method with Fourier Filtering	Proposed Method without Fourier Filtering
MAE	0.1879	0.2169	0.4160	0.0702
RMS	0.0254	0.0276	0.0175	0.0083

## Data Availability

Not applicable.
